# A Very Rare Malformation Affecting the Female Genital System of one *Labronema* Specimen (Dorylaimida, Dorylaimidae)

**DOI:** 10.21307/jofnem-2019-017

**Published:** 2019-04-17

**Authors:** Reyes Peña-Santiago

**Affiliations:** 1 Departamento de Biología Animal, Biología Vegetal y Ecología, Universidad de Jaén, Campus “Las Lagunillas,” Jaén, 23071, Spain

Several kinds of abnormalities or malformations affecting the female genital system of Dorylaimid nematodes have been repeatedly reported in longidorid forms, more occasionally in free-living taxa. These anomalies include total or partial duplication of the system, a didelphic-opisthodelphic condition, total or partial reduction of one genital branch, and the existence of two (or even three) vulvae (see Table 1 for a compendium of previous records). Radivojević (2005) described and discussed with some detail the nature of these anomalies.

**Table 1:** Malformations observed in the female genital system of dorylaims.

Anomaly	Species	Habitat	Country	Reference
Two duplicate systems	*Aporcelaimellus obtusicaudatus*	?	?	Geraert (1963)
	*Eudorylaimus* sp.	?	Spain	Peña-Santiago (1986)
	*Xiphinema diversicaudatum*	Woodland	UK–Scotland	Brown and Coiro (1984)
	*Xiphinema dentatum*	?	Serbia	Radivojević (1991)
Didelphic-opisthodelphic	*Xiphinema coxi coxi*	Alfalfa field	USA–Florida	Cho and Robbins (1990)
Loss of anterior genital branch	*Xiphinema coxi coxi*	Alfalfa field	USA–Florida	Cho and Robbins (1990)
Vulvaless	*Xiphinema dentatum*	?	Serbia	Radivojević (1991)
Two vulvae	*Longidorus danuvii*	*Salix alba*	Poland	Kornobis (2012)
	*Longidorus euonymus*	?	?	Barsi (1994)
	*Longidorus juvenilis*	Vineyard	Slovenia	Sirca et al. (2007)
	*Longidorus laevicapitatus*	Coffee	Sao Tome	Lamberti et al. (1987)
	*Longidorus* sp.	?	?	Jairajpuri and Ahmad (1969)
	*Mesodorylaimus bastiani*	?	?	Loof (1969)
		?	?	Valocká and Sabová (1980)
	*Nygolaimus* sp.	?	?	Jairajpuri and Ahmad (1969)
	*Xiphinema dentatum*	?	Serbia	Radivojević (1991)
	*Xiphinema diversicaudatum*	?	?	Barsi (1994)
		Peach orchard	Czech Republic	Kumari and Decraemer (2009)
	*Xiphinema index*	Fig	Italy	Catalano (1991)
	*Xiphinema turcicum*	?	Yugoslavia	Radivojević (1991)
	*Xiphinema vuittenezi*	Vineyard	Italy	Coiro and Lamberti (1980)
		?	?	Barsi (1994)
		Apple orchard	Czech Republic	Kumari and Decraemer (2006)
		Apple orchard	Czech Republic	Kumari and Decraemer (2009)
Three vulvae	*Mesodorylaimus bastiani*	?	?	Valocká and Sabová (1980)

One female of the genus *Labronema* Thorne, 1939, recently collected in the course of a nematological survey, shows one of the rarest abnormalities so far observed, as the individual lacks both vulva and vagina. The specimen was collected in a grassy and stony soil at 1,800 m.a.s.l. on the mountain of La Pandera, Province of Jaén, Spain. The individual represents a population belonging to a non-described species of the genus *Labronema*, which will be characterized and described in a separate contribution.

**Figure 1:**
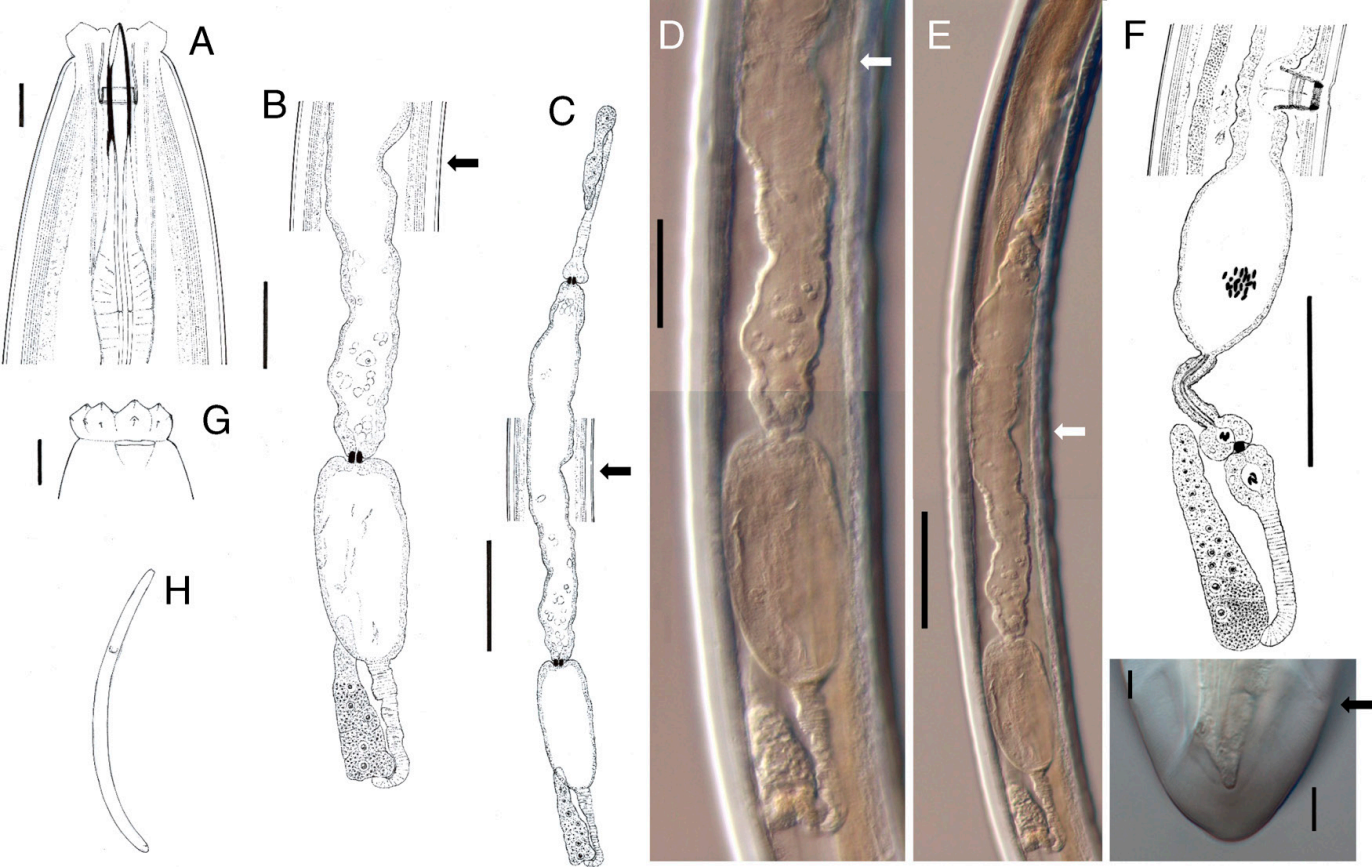
Light micrographs of *Labronema* sp. A–E and G–I, abnormal female; F, normal female. A: anterior region in median lateral view; B: posterior genital branch (arrow pointing at the supposed position of vulva); C, E: genital system (arrow pointing at the supposed position of vulva); F: posterior genital branch; G: lip region in lateral surface view; H: entire body; I: caudal region (arrow pointing at anus). (Scale bars: A, G, I = 10 µm; B, D = 50 µm; C, E, F = 100 µm).

Leaving aside the absence of vulva and vagina, the general morphology (Fig. 1) and morphometry (Table 2) of this female are totally comparable to those observed in other females of the same population. In particular, the length of neither its genital branches (anterior 324 µm or 18% of body length, posterior 362 µm or 20% of body length) nor ovaries (anterior 107, posterior 87 µm) differ from those of normal females (207–368 µm or 13–20% of body length, 43–180 µm, respectively). Nonetheless, some differences are observed in the morphology of genital tract. On one hand, the posterior oviduct appears visibly inflated at its distal part and significantly longer (187 µm) than that observed in normal females (72–147 µm), probably due to fixation process, as the anterior one is comparable to that of normal females (140 and 43–180 µm, respectively). On the other hand, the uteri are apparently simple and tube-like (Fig. 1B,C) (vs complex, tripartite in normal females; Fig. 1F), and sperm cells, always abundant in normal females as the population is bisexual with both females and males nearly equally present, are not found within the genital tracts of the abnormal female. A somewhat similar anomaly was reported by Radivojević (2005) in *Xiphinema dentatum*, in this case also with a significant reduction of both uteri.

**Table 2:** Morphometrics of *Labronema* sp. from Spain. Measurements in µm, except L in mm, and in the form: average ± sd (range).

Character	♀*	11♀♀**
L	1.80	1.81 ± 0.15 (1.56–2.07)
a	22.5	21.5 ± 1.8 (17.8–23.6)
b	3.9	3.8 ± 0.3 (3.3–4.3)
c	56.2	66.1 ± 8.5 (56.4–79.6)
V	?	58.1 ± 0.9 (56.7–59.5)
c’	0.7	0.6 ± 0.1 (0.5–0.8)
Lip region diameter	23	22.2 ± 1.0 (20–23)
Odontostyle length	26	25.1 ± 1.9 (22–28)
Odontophore length	42	40.8 ± 3.8 (33–44)
Neck length	466	472 ± 25 (417–514)
Pharyngeal expansion length	239	241 ± 20 (205–272)
Body diam. at neck base	75	79.2 ± 9.0 (65–98)
Mid-body	80	84.6 ± 8.1 (72–99)
Anus/cloaca	49	43.5 ± 2.9 (40–47)
Distance vulva – anterior end	?	1054 ± 99 (889–1232)
Prerectum length	102	118 ± 24 (84–160)
Rectum/cloaca length	59	59.8 ± 5.0 (52–68)
Tail length	32	27.8 ± 4.0 (24–34)

**Note:** *abnormal female, **normal females.

Vulvaless or *Vul* mutants, which lack not only a vulva but also a vagina, have been generated in experimental studies with *Caenorhabditis elegans* (Horvitz and Sulston, 1980; Ferguson and Horvitz, 1985; Greenwald, 1997; Sternberg, 2005), *Pristionchus pacificus* (Eizinger and Sommer, 1997) and *Oscheius tipulae* (Dichtel-Danjoy and Félix, 2004), an indication that the developmental anomaly noted herein might have a genetic basis. Additionally, environmental conditions might drive the development of vulval anomalies, as demonstrated by Braendle and Félix (2008), in both wild-type and mutant animals of *Caenorhabditis* spp. As mentioned above, the abnormal female herein reported was collected at a moderately high elevation (1,800 m), and might be, for instance, exposed to an extremely stressful environment during its development.
